# A Novel Mutation in Human Androgen Receptor Gene Causing Partial Androgen Insensitivity Syndrome in a Patient Presenting with Gynecomastia at Puberty

**DOI:** 10.4274/jcrpe.2637

**Published:** 2016-06-06

**Authors:** Cemil Koçyiğit, Serdar Sarıtaş, Gönül Çatlı, Hüseyin Onay, Bumin Nuri Dündar

**Affiliations:** 1 Katip Çelebi University Faculty of Medicine, Department of Pediatric Endocrinology, İzmir, Turkey; 2 Tepecik Training and Research Hospital, Clinic of Pediatrics, İzmir, Turkey; 3 Tepecik Training and Research Hospital, Clinic of Pediatric Endocrinology, İzmir, Turkey; 4 Ege University Faculty of Medicine, Department of Medical Genetics, İzmir, Turkey

**Keywords:** Partial androgen insensitivity, gynecomastia, androgen receptor gene

## Abstract

Partial androgen insensitivity syndrome (PAIS) typically presents with micropenis, perineoscrotal hypospadias, and a bifid scrotum with descending or undescending testes and gynecomastia at puberty. It is an X-linked recessive disorder resulting from mutations in the androgen receptor (AR) gene. However, AR gene mutations are found in less than a third of PAIS cases. A 16-year-old boy was admitted with complaints of gynecomastia and sparse facial hair. Family history revealed male relatives from maternal side with similar clinical phenotype. His external genitalia were phenotypically male with pubic hair Tanner stage IV, penoscrotal hypospadias, and a bifid scrotum with bilateral atrophic testes. He had elevated gonadotropins with a normal testosterone level. Chromosome analysis revealed a 46,XY karyotype. Due to the family history suggesting a disorder of X-linked trait, PAIS was considered and molecular analysis of AR gene was performed. DNA sequence analysis revealed a novel hemizygous mutation p.T576I (c.1727C>T) in the AR gene. The diagnosis of PAIS is based upon clinical phenotype and laboratory findings and can be confirmed by detection of a defect in the AR gene. An accurate approach including a detailed family history suggesting an X-linked trait is an important clue for a quick diagnosis.

**WHAT IS ALREADY KNOWN ON THIS TOPIC?**

Partial androgen insensitivity syndrome typically presents with micropenis, perineoscrotal hypospadias, and a bifid scrotum with descending or undescending testes and gynecomastia at puberty. It is an X-linked recessive disorder resulting from mutations in androgen receptor (AR) gene. In approximately 50% of cases, a mutation in AR gene cannot be detected.

**WHAT THIS STUDY ADDS?**

DNA sequence analysis revealed a novel hemizygous mutation p.T576I (c.1727C>T) in the AR gene.

## INTRODUCTION

Androgen insensitivity syndrome (AIS) is the commonest cause of 46,XY disorders of sex development (DSD) and is characterized with defective masculinization of external genitalia in 46,XY individuals despite normal androgen production and metabolism (1). The estimated prevalence of complete AIS (CAIS) ranges from 1:20.400 to 1:99.100 genetic males on the basis of proven molecular diagnosis (2), while partial AIS (PAIS) is at least as common as CAIS (1/130.000) (3). According to the masculinization degree of external genitalia, AIS is divided into three clinical forms including mild AIS (MAIS), PAIS, and CAIS (4).

Patients with CAIS usually present with normal female external genitalia and bilateral inguinal hernias. Individuals who are not diagnosed during childhood are detected after puberty because of primary amenorrhea with a blind vagina and absent uterus. On the other hand, depending on the degree of responsiveness of the external genitalia to androgens, findings in PAIS will show a wide spectrum varying from perineoscrotal hypospadias, bifid scrotum, cryptorchidism and pubertal gynecomastia to extreme inadequate virilization appearing as cliteromegaly and labial fusion (5).

This report describes an adolescent boy with PAIS who presented with gynecomastia at puberty and was found to have a novel mutation in the androgen receptor (AR) gene.

## CASE REPORT

**Figure 1:**
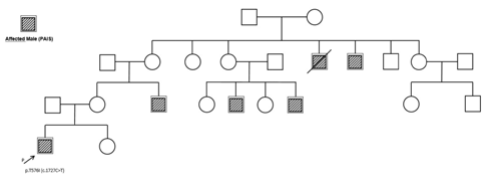
Pedigree

A 16-year-old boy was admitted to our clinic with complaints of gynecomastia and sparse facial hair. He was born at term with a birth weight of 3000 grams. A detailed history revealed operations for bilateral cryptorchidism and penoscrotal hypospadias at ages one and two years, respectively. There was no parental consanguinity. Family history revealed male relatives from the maternal side with similar clinical phenotype, including gynecomastia, hypospadias, sparse facial hair, and infertility (pedigree shown in Figure 1). On physical examination, the patient weighed 94 kg [2.84 standard deviation (SD)] and was 170 cm (-0.56 SD) tall. His external genitalia was phenotypically male with pubic hair Tanner stage IV, penis size 8 cm in length and 2.5 cm in diameter, penoscrotal hypospadias, and a bifid scrotum in which both testes were palpable as 2 mL. He had normal axillary hair and gynecomastia compatible with breast development of Tanner’s stage III. The rest of the physical examination was normal. Hormone levels were as follows: follicle-stimulating hormone (FSH) 42.8 mIU/mL (normal: 1.5-12.4 mIU/mL), luteinizing hormone (LH) 37.4 mIU/mL (normal: 1.7-8.6 mIU/mL), total testosterone (T) 419 ng/dL (normal: 180-763 ng/dL), estradiol 30.5 pg/mL (normal: 7.6-42 pg/mL), beta-human chorionic gonadotropin (β-hCG) 0.73 mIU/mL (normal: 0-2 mIU/mL), and alpha-fetoprotein 2.78 ng/mL (normal: 0-7 ng/mL). Scrotal ultrasound revealed that both testes were atrophic and were 28x10x17 mm (left) and 12x14x27 mm (right) in size. Chromosome analysis revealed a 46,XY karyotype. Due to the family history suggesting a disorder of X-linked trait, PAIS was considered and molecular analysis of AR gene was performed. DNA sequence analysis revealed a novel mutation hemizygous p.T576I (c.1727C>T) in the AR gene. Due to the presence of atrophic testes and increased risk of germ cell tumor development, bilateral gonadal biopsy was recommended. However, the patient and his family did not accept the procedure. Therefore, the patient is still being followed-up by physical examination and testis ultrasonography on a six-monthly basis.

### Molecular Analysis

To investigate the etiology of proband’s PAIS, after getting informed consent from the parents, genomic DNA was extracted from peripheral EDTA anticoagulant whole blood using the MagNA Pure LC automated system (Roche Applied Science, Manheim, Germany) following the manufacturer’s instructions. The polymerase chain reaction fragments were sequenced by Illumina MiSeq system using V2 chemistry (Illumina, Ca, USA). Sequencing results were analyzed using IGV software (http://www.broadinstitute.org/igv/).

**Figure 2:**
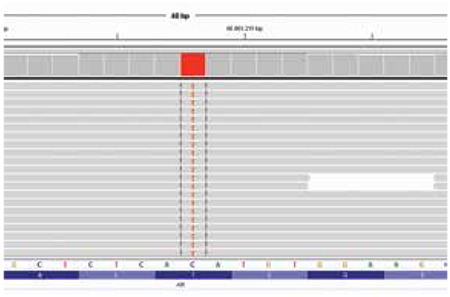
Hemizygous p.T576I (c.1727C>T) mutation detected with MiSeq NGS system

A hemizygous mutation p.T576I (c.1727C>T), which had not previously been reported, was identified in the AR gene (Figure 2). Analysis of this novel mutation by bioinformatic tools that examine functional effects of single nucleotide variants in humans [Mutation Taster (http://www.mutationtaster.org)] predicted the variant p.T576I (c.1727C>T) to be disease causing.

## DISCUSSION

The AIS describes a spectrum of disorders where the degree of receptor insensitivity may vary from minimal to complete insensitivity. In case of MAIS, the individual is phenotypically male with sterility, azoospermia, and gynecomastia without genital abnormalities. The other end of the spectrum comprises XY individuals with CAIS who phenotypically appear as tall females with well-developed breasts, blind vagina, and absent or scanty pubic and axillary hair (6). The typical phenotype in PAIS is micropenis, perineoscrotal hypospadias, and a bifid scrotum with descending or undescending testes, and gynecomastia at puberty. In the present patient, penoscrotal hypospadias and bilateral cryptorchidism and gynecomastia at puberty were considered as the clinical findings of PAIS.

In the differential diagnosis of PAIS, 5α-reductase deficiency, partial gonadal dysgenesis (due to mutations in SF1, SRY, WT1 etc.), and testosterone biosynthesis defects need to be considered. If the karyotype is 46,XY, then serum testosterone and dihydrotestosterone levels are helpful for the differential diagnosis. Normal or elevated levels of testosterone and dihydrotestosterone are essential to exclude androgen biosynthesis defects. Nevertheless, hCG stimulation test may be necessary to differantiate partial gonadal dysgenesis in prepubertal children (5). While an elevated or normal testosterone level suggests PAIS or 5hile an elevated ordeficiency, a low testosterone level is indicative for partial or complete gonadal dysgenesis (7). Serum anti-Müllerian hormone (AMH) level is another useful tool in the differential diagnosis of DSD. AMH is synthesized from Sertoli cells and in healthy males, its level decreases at puberty due to the stimulation of AR on Sertoli cells. Serum AMH levels are low in gonadal disgenesis due to aberrant Sertoli cell development and tend to be abnormally high in CAIS because of the lack of intact ARs on Sertoli cells (8,9,10). In our case, due to financial difficulties, we were unable to measure serum AMH level.

In cases with AIS, high levels of testosterone, a substrate for aromatase activity, result in substantial amounts of estrogens, which are responsible for breast development at puberty. However, due to functional AR activity, gynecomastia has not been reported in cases with 5α-reductase type 2 deficiency and partial gonadal dysgenesis (5,7). In the present patient, normal testosterone level and the presence of gynecomastia made us consider PAIS. Although the size of the testes is usually normal in PAIS, PAIS cases with atrophic testes have also been reported (11,12). Similar to the previous reports, in our patient, delayed orchiopexy might have led to atrophy in the testes.

The hormonal profile is similar in individuals with CAIS and PAIS. At birth, levels of testosterone and LH remain high or slightly above the normal range for males. During puberty, individuals with PAIS maintain normal or slightly elevated testosterone and LH levels (7). Estradiol, which is derived from peripheral conversion of testosterone and from testicular secretion, tends to be in the normal female range (8). Serum FSH levels are reported to be normal in AIS (13). However, serum FSH level is high in our patient, reflecting the damage to the seminiferous tubules possibly due to delayed orchiopexy of intraabdominal testes.

AIS is an X-linked recessive disorder resulting from mutations in the AR gene (14). While the vast majority of CAIS cases (90-95%) are attributable to AR mutations, less than a third of cases with a phenotype consistent with PAIS are associated with AR mutations (15), To date, more than 800 mutations have been identified in the AR gene (7,16,17). In the current patient, detailed family history revealed male relatives from the maternal side with similar clinical phenotype suggesting an X-linked trait, which was an important clue for the diagnosis of PAIS. PAIS has a broad heterogeneity in phenotypic expression, which is partly explained by different AR defects. However, individuals with same mutations may exhibit widely variable phenotypes both within and between affected families (16). As a result, there is no definite relationship between phenotype and genotype in PAIS, suggesting that other factors are contributing to the degree of masculinization (18). The present patient was identified to have a novel hemizygous mutation p.T576I (c.1727C>T) in the human AR gene. However, a limitation of this report is that we could not perform molecular analysis of other affected family members and therefore cannot comment on the relationship between genotype and phenotype.

Gender decision, genitoplasty, timing of gonadectomy (due to cancer risk), hormone replacement therapy, genetic counseling, and psychological support comprise the basis of management in PAIS. The majority of the patients with PAIS are reared as males (19). When the external genitalia is female, treatment is similar to that for CAIS, except that gonadectomy is recommended before puberty to avoid the physical and emotional discomfort of pubertal virilization. On the other hand, if the patient had a penis, albeit small, a male sex is assigned, and the individual may have to wait until puberty for the clinical picture to manifest more clearly by a lack of male secondary characteristics and the development of gynecomastia (7). The present patient had a phenotypically male external genitalia with a penis size 8 cm in length and 2.5 cm in diameter and penoscrotal hypospadias. He had been reared as male and psychiatric evaluation revealed a male gender identity at the age of sixteen.

Patients with PAIS are under the risk of malignancy which mostly are gonadoblastoma or dysgerminoma. Gonadal tumor risk is 0.8-2% during the prepubertal period and rises up to 30% during late adulthood. Risk of malignancy is low before the age of 25 years and more frequent between 30 and 50 years (20). The risk of type 2 germ cell tumors is higher in PAIS than in CAIS, with a suggested incidence of 15% and even higher (~50%) if the testes are not scrotal in position (19,21). Due to the high risk of malignancy, gonadectomy at the time of diagnosis is the current recommendation for PAIS if presenting with undescended testes (non-scrotal). However, it was also reported that gonadectomy might be delayed until a stable gender identity has been established (21). Many authors recommend gonadectomy after puberty to achieve adequate bone mineralization and body maturation. However, some researchers recommend biopsy at the end of the pubertal age (17-24 years) and gonadectomy if a premalignant lesion and/or carcinoma in situ are detected (7,22,23). Our patient was under the risk of a gonadal tumor as he had a history of cryptorchidism and delayed orchiopexy. A testicular biopsy was recommended but could not be performed since the parents and the patient refused the procedure.

In conclusion, PAIS constitutes one of the most common causes of 46,XY DSD. The diagnosis is based upon clinical phenotype and laboratory findings, and can be confirmed by detection of a defect in the AR gene. An accurate approach including a detailed family history suggesting an X-linked trait is an important clue for a quick diagnosis.

## Ethics

Informed Consent: It was taken.

Peer-review: External peer-reviewed.
